# Aromatase deficiency in transplanted bone marrow cells improves vertebral trabecular bone quantity, connectivity, and mineralization and decreases cortical porosity in murine bone marrow transplant recipients

**DOI:** 10.1371/journal.pone.0296390

**Published:** 2024-02-05

**Authors:** Katie Rubitschung, Amber Sherwood, Rasesh Kapadia, Yin Xi, Asghar Hajibeigi, Katya B. Rubinow, Joseph E. Zerwekh, Orhan K. Öz

**Affiliations:** 1 Department of Radiology, University of Texas Southwestern Medical Center, Dallas, Texas, United States of America; 2 Scanco USA Incorporated, Wayne, Pennsylvania, United States of America; 3 Division of Metabolism, Endocrinology, and Nutrition, University of Washington Medicine Diabetes Institute, Seattle, Washington, United States of America; 4 Charles and Jane Pak Center for Mineral Metabolism and Clinical Research, UT Southwestern Medical Center, Dallas, Texas, United States of America; Nanjing Medical University, CHINA

## Abstract

Estradiol is an important regulator of bone accumulation and maintenance. Circulating estrogens are primarily produced by the gonads. Aromatase, the enzyme responsible for the conversion of androgens to estrogen, is expressed by bone marrow cells (BMCs) of both hematopoietic and nonhematopoietic origin. While the significance of gonad-derived estradiol to bone health has been investigated, there is limited understanding regarding the relative contribution of BMC derived estrogens to bone metabolism. To elucidate the role of BMC derived estrogens in male bone, irradiated wild-type C57BL/6J mice received bone marrow cells transplanted from either WT (WT(WT)) or aromatase-deficient (WT(ArKO)) mice. MicroCT was acquired on lumbar vertebra to assess bone quantity and quality. WT(ArKO) animals had greater trabecular bone volume (BV/TV p = 0.002), with a higher trabecular number (p = 0.008), connectivity density (*p* = 0.017), and bone mineral content (*p* = 0.004). In cortical bone, WT(ArKO) animals exhibited smaller cortical pores and lower cortical porosity (p = 0.02). Static histomorphometry revealed fewer osteoclasts per bone surface (Oc.S/BS%), osteoclasts on the erosion surface (ES(Oc+)/BS, p = 0.04) and low number of osteoclasts per bone perimeter (N.Oc/B.Pm, *p* = 0.01) in WT(ArKO). Osteoblast-associated parameters in WT(ArKO) were lower but not statistically different from WT(WT). Dynamic histomorphometry suggested similar bone formation indices’ patterns with lower mean values in mineral apposition rate, label separation, and BFR/BS in WT(ArKO) animals. *Ex vivo* bone cell differentiation assays demonstrated relative decreased osteoblast differentiation and ability to form mineralized nodules. This study demonstrates a role of local 17β-estradiol production by BMCs for regulating the quantity and quality of bone in male mice. Underlying *in vivo* cellular and molecular mechanisms require further study.

## Introduction

Aromatase is an oxidoreductase that is responsible for the conversion of androgens into estrogens. Plasma estradiol levels in males are influenced by estradiol production from testosterone, which is largely formed in the testes. However, plasma estradiol levels do not reflect tissue-level estrogen activity as extra-gonadal aromatization of androgens to form estrogens is known to occur [1, 2]. In human males, loss-of-function mutations of the aromatase gene *CYP19A1* or the gene encoding estrogen receptor-α (ERα) lead to estrogen deficiency or resistance. Since estrogen-regulated epiphyseal fusion does not occur, these individuals have prolonged linear bone growth and delayed bone maturation as well as osteopenia [3]. The global aromatase knockout (ArKO) mouse model recapitulates many phenotypes seen in human males and has been used to demonstrate the importance of systemic estrogens for bone health. Male ArKO mice have an osteopenic skeletal phenotype with low bone turnover [4], increased adiposity [5, 6], and insulin resistance [7].

Mesenchymal stem cells (MSCs) can differentiate into many cell types including osteoblasts, osteoclasts, adipocytes, chondrocytes, myocytes, endothelial cells, and fibroblasts. Aromatase expression has been reported in human and murine bone cells such as osteoblasts and osteoclasts [8, 9]. Hematopoietic stem cells (HSCs) of red bone marrow give rise to cells of the myeloid or lymphoid lineages [10]. Mature cells of myeloid lineage include erythrocytes and immune cells such as monocytes, macrophages, neutrophils, basophils, eosinophils, and dendritic cells [11], while T cells, B cells, and natural killer cells are derived from lymphoid precursors. A number of HSC descendants from both human and murine cells express aromatase, including T lymphocytes [12], B lymphocytes [13, 14], and macrophages [15].

Previous studies have shed some light on the role of local estrogen signaling in hematopoietic HSC and MSC progeny relative contribution to bone turnover and mass. Gustafsson et al. used a female murine model lacking ERα expression specifically in T lymphocytes (Lck-ERα^−/−^) to determine if the estrogenic regulation of bone turnover is dependent on ERα expression in T lymphocytes. Ovariectomy resulted in similar bone mineral density (BMD) decreases between Lck-ERα^−/−^ and ERα^flox/flox^ control mice. Additionally, estrogen treatment of ovariectomized Lck-ERα^−/−^ and ERα^flox/flox^ controls resulted in similar BMD increase [12]. Together, these results indicate that ERα expression in T lymphocytes is dispensable for estrogenic regulation of bone. A bone marrow transplant study of ovariectomized WT and ERαKO female mice found that bone mass improved with exogenous estrogen supplementation in WT animals which received either WT or ERαKO bone marrow, but bone mass could not be recovered in ERαKO mice regardless of donor marrow ERα status. While the bone mass of both WT recipient groups was increased, estradiol treated WT(WT) females exhibited a greater increase in cortical and trabecular bone mass than their WT(ERαKO) counterparts. This data suggests that, although ERα is not required in hematopoietic cells for estrogenic bone signaling, estrogen regulation of bone mass is enhanced by the presence of ERα in bone marrow cells [16]. Although these studies provided valuable insights regarding the contribution of estrogen signaling through ERα on bone maintenance in the female skeleton, similar studies have not been performed on male mice or in states of local loss of estrogen synthesis (estrogen deficiency).

To study the role of bone marrow derived estrogens in local bone turnover and maintenance in male mice, cells derived from aromatase deficient (ArKO) or WT, mice were transplanted into WT recipient animals to generate WT(ArKO) and WT(WT) animals. The aim of this study was to determine the importance of bone marrow derived estrogens for maintenance of bone mass, structure, and turnover. This aim was achieved using microCT and histomorphometry. Potential cellular mechanisms underlying the observed phenotypes, were explored using *ex vivo* osteoblast and osteoclast differentiation assays starting from bone marrow residing precursors.

## Materials and methods

### Animals

Animals and the bone marrow transplant procedure are described in our previous publication [17]. Briefly, heterozygous congenic C57BL/6J aromatase-deficient male and female mice were generated as previously described [5, 18] and bred to generate wild-type (WT) and aromatase-deficient (ArKO) littermates. Congenic C57BL/6J breeders were generated at the University of Texas Southwestern Medical Center (UTSW) by the senior author (OKÖ). Harem breeding pairs were genotyped and provided to the Rubinow lab for metabolic studies after bone marrow transplant as described [17]. All breeding procedures were approved by the UTSW IACUC.

Eight to ten-week-old male bone marrow donors (n = 4 per genotype) were sacrificed by CO_2_ inhalation and exsanguination. After harvest, femurs and tibias were washed once in ethanol, and three times in phosphate-buffered saline (PBS). The bone was cut at the distal growth plate, placed in 600 μL tubes punctured with an 18” needle and placed within a 1.5 mL tube. Centrifugation occurred at 10*g* for 8 seconds. The bone marrow was collected in the 1.5 mL tube, resuspended in 1 mL of red blood cell lysis buffer (Sigma-Aldrich; St. Louis, MO), and pooled with donor mice of the same genotype in 50 mL tubes. The lysis reaction was quenched using 3–5 mL of PBS. The samples were subsequently centrifuged at 400*g* for 5 minutes. After aspirating off the supernatant, the cell pellet was resuspended in 1% PBS to a final concentration of 23.8 x 10^6^ cells/mL and aliquoted into syringes for bone marrow transplant. In a separate analysis, 10-week-old ArKO and WT males were euthanized by cervical dislocation under isoflurane anesthesia and the marrow was harvested from the long bones of each animal. BMC viability was determined via trypan blue exclusion on 24 bones from WT mice and 18 bones from ArKO mice.

Male bone marrow transplant recipient mice were WT C57BL/6J mice purchased from Jackson Laboratory (Bar Harbor, ME; strain #000664). All mice were maintained on a 12h light-dark cycle and regular chow diet (D12450H, Research Diets, Inc; New Brunswick, NJ, USA) with ad libitum access to food and water. Recipients were sacrificed by exsanguination and cervical dislocation under isoflurane 24 weeks after transplantation. The bone marrow transplantation procedure was carried out in adherence with the recommendations in the Guide for the Care and Use of Laboratory Animals of the National Institutes of Health. All transplantation related procedures were approved in advance by the University of Washington Institutional Animal Care and Use Committee (IACUC, protocol #4369–01) and all efforts were made to minimize suffering.

Following 4 weeks of acclimation, transplant recipients were irradiated with 10 Gy. One day after irradiation, surgeries were performed under isoflurane anesthesia. Transplant recipients received 7 x 10^6^ cells (300 μL injection) by retro-orbital injection. Bone marrow transplant recipients were 9-week-old WT mice with marrow cells donated from 8 to 10-week-old WT (henceforth referred to as WT(WT) mice) or ArKO littermate donors (WT(ArKO) mice).

#### Post-transplant care, skeletal labeling & tissue harvest

Following bone marrow transplant, recipient mice received neomycin (2 mg/mL) in drinking water for 2 weeks post-irradiation. Transplant recipients were monitored 3–4 times weekly for 4 weeks following irradiation until marrow engraftment occurred. Bone marrow engraftment was verified through genotyping of circulating immune cells. Mice were monitored for any indicators of poor health and any concerning signs or behaviors were reported to veterinary medicine.

Twenty-eight weeks after transplant, bone marrow recipients were sacrificed, and the lumbar spine was harvested for microCT analysis and histology. Spines were fixed in 10% neutral buffered formalin for 24 hours and stored in 70% ethanol.

To label the skeleton, animals underwent intra-peritoneal injection of the inert fluorochromes alizarin complexone dihydrate (25 mg/kg; Sigma-Aldrich; St. Louis, MO) and calcein green (5 mg/kg body weight, Honeywell Fluka; Mexico City, MEX), at 7 and 2 days prior to sacrifice, respectively.

### MicroCT

To assess the 3D structure of cortical and trabecular bone, microCT scanning was conducted on the L4 vertebral bodies using a Scanco Medical μCT 40 (Bruettisellen, Switzerland). The scans were acquired at an x-ray energy level of 55 kV using a current of 145 μA and an isotropic voxel size of 6 μm. A series of 600–650 slices were obtained covering a height of approximately 3.5 to 3.8 mm per vertebrae.

Morphometric analysis was conducted on the images using the accompanying Scanco software suite. The trabecular volume of interest, encompassing about 350 slices of the L4 vertebral body, was defined using a combination of manual and an interpolation process. The cortical volume of interest encompassed 200 slices of the L4 vertebral body and was automatically contoured using a script provided by Scanco Medical and adapted from Buie et al. [19]. Three-dimensional analysis of the trabecular compartment was conducted using a global threshold of 260 per mille (‰) and a gauss filter (sigma 0.8; support 1). Three-dimensional analysis of the cortical compartment was conducted using a lower threshold of 200, upper threshold of 700, and gauss filter (sigma 0.8; support 2).

To detect differences between groups in cortical thickness distribution, the anterior view of each μCT image was analyzed by splitting a color scale into 10 equal segments ranging from dark blue (thin) to red (thick). The number of pixels per color were quantified in GNU Image Manipulation Program (GIMP 2.10.30) and graphed.

For cortical porosity measurements, 150 slices of the L4 vertebral body were defined using a combination of manual and interpolation procedures. Porosity was measured on the anterior cortical wall with an ROI bounded between the transverse spinal processes. Analysis was conducted using a lower threshold of 270 and upper threshold of 1000, and gauss filter (sigma 0.5; support 1).

### Static histomorphometry

#### Tissue processing

Following removal of overlying musculature, spine specimen histology was performed. Spinal columns were embedded in methyl methacrylate resin and sectioned at 5 μm thickness by the Research Histology Core Laboratory at MD Anderson Cancer Center. Staining was performed by the Bone Histomorphometry Core Laboratory at MD Anderson Cancer Center for osteoblasts and osteoid using a modified Goldner’s Trichrome (Weigert’s Hematoxylin, Acid fuchsin-ponceau, phosphomolybdic acid-phosphotungstic acid-Orange G, aniline blue.) The TRAP enzymatic stain was performed for visualization of osteoclasts (0.2M Acetate buffer, Napthol AS-MX phosphate, Fast Red TR salt hemi salt).

#### Static histomorphometry evaluation parameters and analysis

For bone formation parameters, osteoblasts were counted only if they retained their cuboidal shape along the trabecular bone. Lining cells were excluded. Osteoid was measured where the acid fuchsin-ponceau stained along the bone surface. As osteoclasts also stain red with the modified Goldner’s Trichrome, morphology was carefully considered to ensure osteoclasts weren’t mistaken as osteoid deposits. Data was provided for both bone surface with osteoblasts and bone surface with osteoblasts and osteoid.

For resorption parameters, osteoclasts were counted if they stained red with the TRAP stain and were in contact with the bone surface. Stained osteoclasts that were in the marrow space away from trabecular bone were excluded. If there was staining along the bone surface, but no nuclei were present, the surface was classified as erosion surface and included in the final numerical data. Erosion was only evident in areas with the presence of an osteoclast on the bone surface.

Bioquant OSTEO II software (BIOQUANT OSTEO II 2021 Version 21.5.6, Nashville, TN, United States) was used for analysis. The L4 vertebrae was analyzed, excluding the region nearest the vertebral wall, and measuring in 150μm to avoid the primary spongiosa and the spinal processes. In one instance where the L4 vertebra was damaged, L3 was analyzed.

### Dynamic histomorphometry

Confocal images were captured at 20X magnification using a Zeiss LSM 880 confocal microscope system equipped with 488nm and 561nm lasers to capture calcein and alizarin fluorescence, respectively. Images were acquired using the tile scanning and stitching feature of the Zeiss 3.5 Blue edition software.

Using Fiji (ImageJ v1.53q), the vertical and horizontal measurements of the marrow cavity were obtained [20]. The region of interest was generated by selecting the centermost 75% of the marrow area. To ensure consistent measurements between samples, the scalebar of a single image was used to globally calibrate all images. The total bone surface was directly measured by drawing a contour of all bone within the region of interest using the freehand line tool. The double labelled surfaces and single labelled surfaces were directly measured using the freehand line tool to generate a contour of each surface, respectively. The average label separation of a double labelled surface was measured by calculating the average length of 3 individual measurements made using the straight-line tool. The MS/BS, MAR, and BFR/BS parameters were calculated using the formulas approved by the ASBMR Histomorphometry Nomenclature Committee [21].

#### *Ex vivo* bone marrow osteoprogenitor assays osteoblast assays

Bone marrow cells were harvested from both femurs of WT and ArKO mice by excising the ends of the disarticulated femurs, placing the bone, proximal end down, in 1 ml of Hank’s buffered saline solution and centrifuging at 10,000*g* for 2 minutes to recover precursor cells. Cells were pooled from 2–3 mice/genotype. All cultured cells were maintained in a humidified atmosphere of 5% CO_2_ and one-half of the medium was replaced every 3 days. Assays were performed 3 times each.

#### CFU-F and CFU-Ob assays

Quantitation of bone marrow osteoprogenitors was determined according to the method of Jilka et al. with minor variations [22]. Briefly, osteoprogenitor cultures were established in 6-well plates at a plating density of 1.5 x 10^6^ cells/well or 2.5 x 10^5^ cells/well for CFU-F (early osteoblastic progenitors) and CFU-Ob (committed osteoblastic precursors), respectively. Osteoprogenitor cells were maintained in phenol red free α-MEM containing 15% preselected FBS, 50 μM ascorbic acid, and 10 mM β-glycerophosphate. For the determination of CFU-F, cells were cultured for 7 days and then stained for alkaline phosphatase and counterstained with hematoxylin (Sigma Diagnostics Alkaline Phosphatase Kit 86-R). Colonies of cells containing a minimum of 25 cells were designated as CFU-F. Mineralized nodule formation (CFU-Ob) was allowed to proceed by culturing the cells to confluence (usually total of 14 to 21 days). Following fixation in 50% ethanol and 18% formaldehyde mixture, mineralized nodules of CFU-Ob plates were stained with 40mM Alizarin Red (S) pH 10.8 dissolved in dH_2_O (MP Biomedicals) [23]. Quantification of CFU-F and CFU-Ob cultures was conducted by counting macroscopic colonies and Alizarin Red (S) positive nodules, respectively.

#### Osteoclast differentiation assays

Osteoclast differentiation assays were produced according to the method of Takahashi et al. [24]. Briefly, osteoclast cultures were established in 24-well plates at a plating density of 1.5 x 10^6^ cells/well. Osteoclast progenitors were induced to differentiate in phenol free α-MEM containing 10% FBS, penicillin/streptomycin, containing 5 ng/mL of M-CSF and 50 ng/mL of RANKL. CFU-Oc plates were cultured for a total of 6–8 days. CFU-Oc plates were quantified by manually counting TRAP positive, multinucleated cells. TRAP-positive cells with 3 or more nuclei were considered osteoclasts.

#### 17β-estradiol (E2) quantification in bone marrow cells

Bone marrow cells were harvested from femurs, as described above, from eight-to nine-week-old WT (n = 4) and ArKO male mice (n = 2). To determine E2 levels in BMCs, cell lysates were made using RIPA buffer. Bone marrow estradiol levels were determined using a mouse Estradiol Kit (ab285237, Abcam) according to the manufacturer’s instructions.

### Statistical analysis

For microCT data, multivariate analysis of variance (MANOVA) was used to test the overall difference in bone measurements between WT(ArKO) and WT(WT) mice. Hotelling’s T statistic was used for hypothesis testing. Univariate t-tests were also performed on each measurement. Pairwise correlations among the measurements were assessed by Pearson correlation coefficients. P values were adjusted by false discovery rate. All analyses were done in SAS 9.4 (SAS institute Inc., Cary, NC). Data is reported as mean ± SD.

For histomorphometry data, the Wilcoxon rank sum test was used to test the overall difference in bone measurements between WT(ArKO) and WT(WT) mice. For ex vivo osteoprogenitor and osteoclast differentiation assays, data normality was tested by the Wilk-Shapiro test. Significance of differences between groups was determined by Student’s t-test for normally distributed data. When data was not normally distributed, the Kruskal-Wallis test was used for analysis of variance among the experimental groups. Specific group-to-group comparisons were performed by the Mann-Whitney test for non-normally distributed data. In all cases, p<0.05 was considered statistically significant. Data is reported as mean ± SD. All graphs were generated using Graphpad Prism 10.0.0 software.

## Results

### Compared to WT bone marrow cells, cells from ArKO mice show no difference in viability and lower but statistically insignificant estradiol levels

There were no differences in viability of BMCs of WT vs. ArKO donors (88.6 ± 1.0% vs. 86.1 ± 2.5%, p = 0.13). To compare 17β-estradiol concentrations in bone marrow cells that were used to reconstitute the irradiated WT mice used in the transplantation study, bone marrow cells were harvested from femurs of global ArKO (n = 2) and WT (n = 4) animals. Overall, the levels were very low in both genotypes. Although ArKO animals had generally lower concentrations of 17β-estradiol, there was no statistically significant difference between WT and ArKO 17β-Estradiol concentrations (12.05 ± 4.35 ng/L vs. 6.21 ± 2.61 ng/L, *p* = 0.09), likely due to wide standard deviation.

### Transplantation of aromatase deficient bone marrow cells is associated with higher trabecular bone volume, trabecular number, and connectivity on microCT

**Fig 1:**
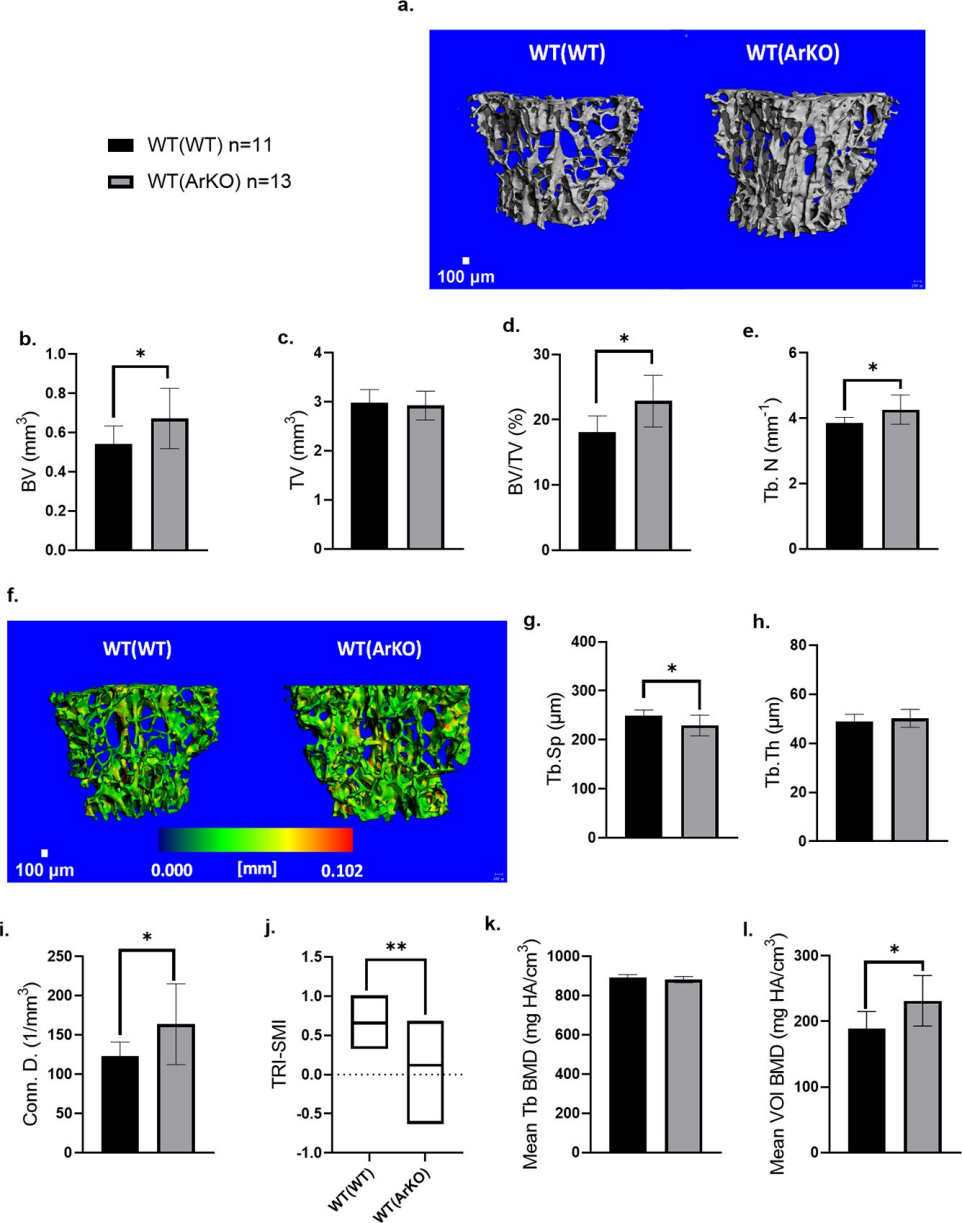
MicroCT analysis demonstrates higher trabecular bone quantity and better quality (connectivity) in WT(ArKO) mice compared to WT(WT) mice. Representative 3-D rendering of the L4 trabecular bone compartment (a). Trabecular bone morphometry (b-j) and density (k-l) measurements were made after segmentation of the trabecular compartment in microCT images. WT(ArKO) animals had increased bone volume (BV) (b), comparable total tissue volume (TV) (c), and increased bone volume fraction (BV/TV) (d) compared to WT(WT) animals. Higher trabecular number (Tb.N) (e) is seen in the trabecular thickness maps (f). Reduced trabecular spacing (Tb.Sp) (g), comparable trabecular thickness (Tb.Th) (h), and improved connectivity density (Conn. D.) (i) are seen in WT(ArKO) animals. The structural model index (TRI-SMI) value for the WT(ArKO) animals indicates a parallel plate like appearance compared to the WT(WT) which indicates a spindle like trabecular appearance (j). While mean trabecular BMD (Mean Tb BMD) (k) was not different, the mean volume of interest BMD (Mean VOI BMD) (l) was notably higher in the WT(ArKO) animals. Data are expressed as mean ± SD. Scale bar = 100μm. *p ≤ 0.05, **p ≤ 0.01. (WT(WT) n = 11 animals, WT(ArKO) n = 13 animals).

Compared to WT(WT), WT(ArKO) mice exhibited significantly higher trabecular bone volume (Fig 1A & 1B, 0.67 mm^3^ vs 0.54 mm^3^, *p* = 0.03), but similar total tissue volume (Fig 1C, *p* = 0.61), resulting in a larger trabecular bone volume fraction (Fig 1D, 22.9% vs 18.1%, *p* = 0.01). Trabecular microarchitecture in WT(ArKO) mice was improved as demonstrated by an increased trabecular number (Fig 1E, 4.26 mm^-1^ vs 3.85 mm^-1^, *p* = 0.02), decreased trabecular spacing (Fig 1G, 229.38 μm vs 249.81 μm, *p* = 0.02) and increased connectivity (Fig 1I, 163.66 /mm^3^ vs 122.83 /mm^3^, *p* = 0.03). Trabecular spacing was significantly decreased in WT(ArKO) mice (Fig 1G, 229.38 μm vs 249.81 μm *p* = 0.02). Interestingly, trabecular thickness was not different between WT(WT) recipients and WT(ArKO) mice (Fig 1F & 1H, *p* = 0.38). In addition, the structure model index between the two groups were significantly different (Fig 1J, 0.119 vs 0.658, *p* = 0.003), with WT(WT) mice displaying trabeculae more closely resembling cylindrical rods and WT(ArKO) mice expressing trabeculae with a more parallel plate-like structure. Average bone mineral density for the whole volume of interest was significantly higher in the WT(ArKO) group (231.46 mm HA/cm^3^) compared to the WT(WT) control group (188.66 mm HA/cm^3^) (Fig 1L, *p* = 0.02); however, no differences were seen in mean trabecular bone mineral density (Fig 1K, *p* = 0.13).

### WT(ArKO) mice show decreased cortical porosity and smaller diameter cortical pores with similar cortical bone mass mice

**Fig 2:**
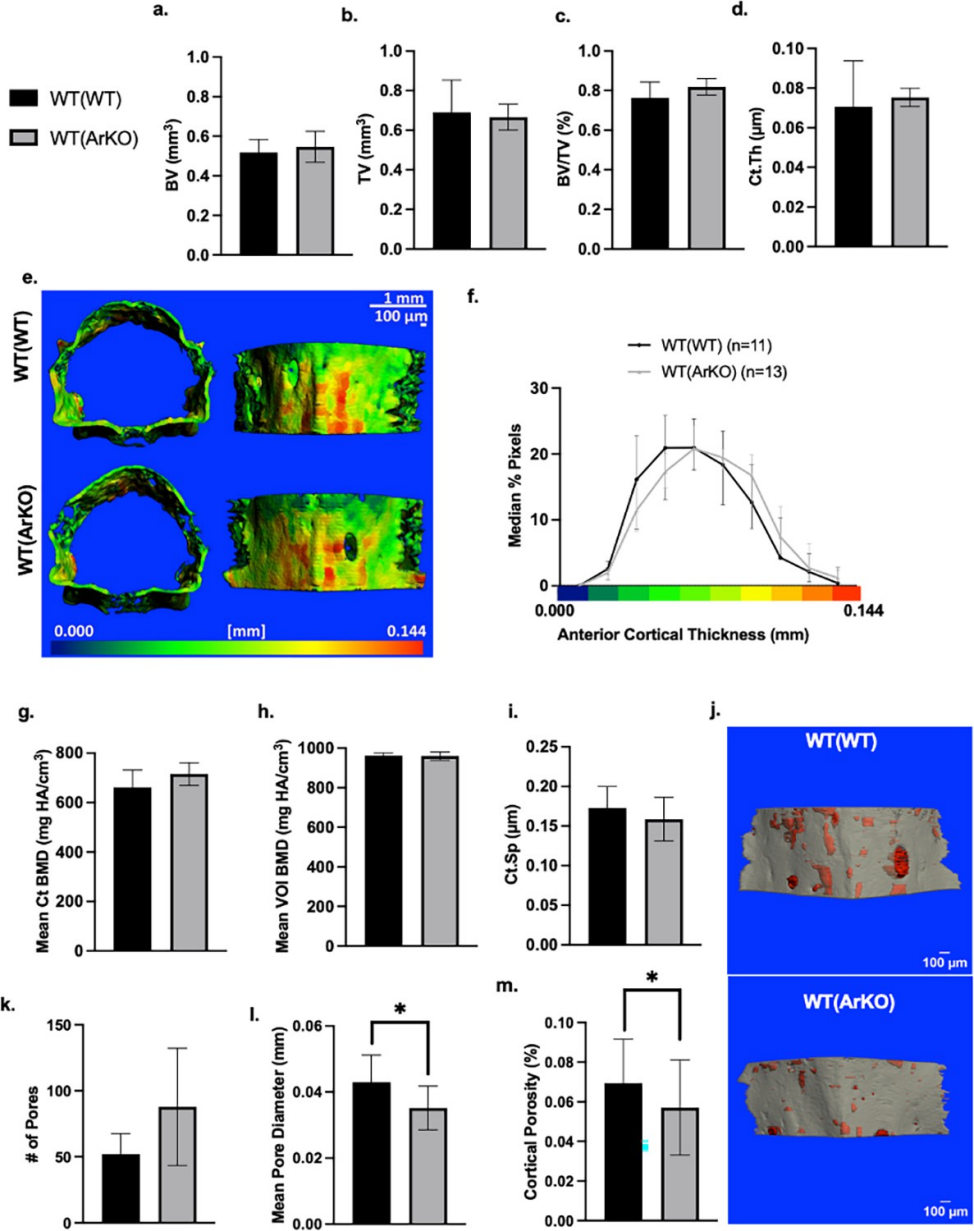
MicroCT analysis indicates similar cortical bone quantity and quality of WT(ArKO) mice and smaller cortical pores and lower porosity compared to WT(WT) mice. MicroCT analysis of L4 vertebral cortical bone showed similar (a) bone volume, (b) total volume, and (c) bone volume fraction. The mean overall cortical thickness was not different by quantification (Ct.Th) (d). Ventral cortical surface thickness maps (e) were quantified pixel-wise to show a right-shift in thickness distribution in WT(ArKO) mice (f). No differences in the mean cortical bone mineral density (g), mean volume of interest (h), or cortical spacing (i) were detected. MicroCT porosity analysis of the anterior face of the L4 vertebral cortical bone (j) showed comparable pore number (k), but pore diameter (l) and cortical porosity (m) appeared significantly lower in WT(ArKO) animals. Data are expressed as mean ± SD. Scale bar = 100μm. *p ≤ 0.05. (WT(WT) n = 11 animals, WT(ArKO) n = 13 animals).

WT(WT) and WT(ArKO) mice exhibited similar cortical bone parameters as measured by microCT, including cortical bone volume (Fig 2A, *p* = 0.53), total tissue volume (Fig 2B, *p* = 0.78), and bone volume fraction (Fig 2C, *p* = 0.09). Overall cortical thickness was not statistically different (Fig 2D, *p* = 0.09). However, to detect possible anisotropic differences between groups, the anterior cortical compartment thickness map was assessed by histogram analysis of pseudo-colored images (Fig 2E & 2F).

While there was a shift toward higher cortical thickness for the WT(ArKO) curve relative to WT(WT), the difference was not statistically significant. Bone mineral content of the cortical bone (Fig 2G, *p* = 0.09) and the whole volume of interest (Fig 2H, *p* = 0.83) were comparable and no differences in cortical spacing were found (Fig 2I, *p* = 0.43). On investigation of cortical porosity (Fig 2J), the number of pores was not statistically different (Fig 2K), however, WT(ArKO) animals had pores with smaller diameter (Fig 2L, 0.03 mm vs. 0.04 mm, *p* = 0.02) and reduced cortical porosity (Fig 2M, 0.05% vs. 0.07%, *p* = 0.02) compared to WT(WT) animals.

### WT(ArKO) mice have fewer osteoblasts per bone perimeter

**Fig 3:**
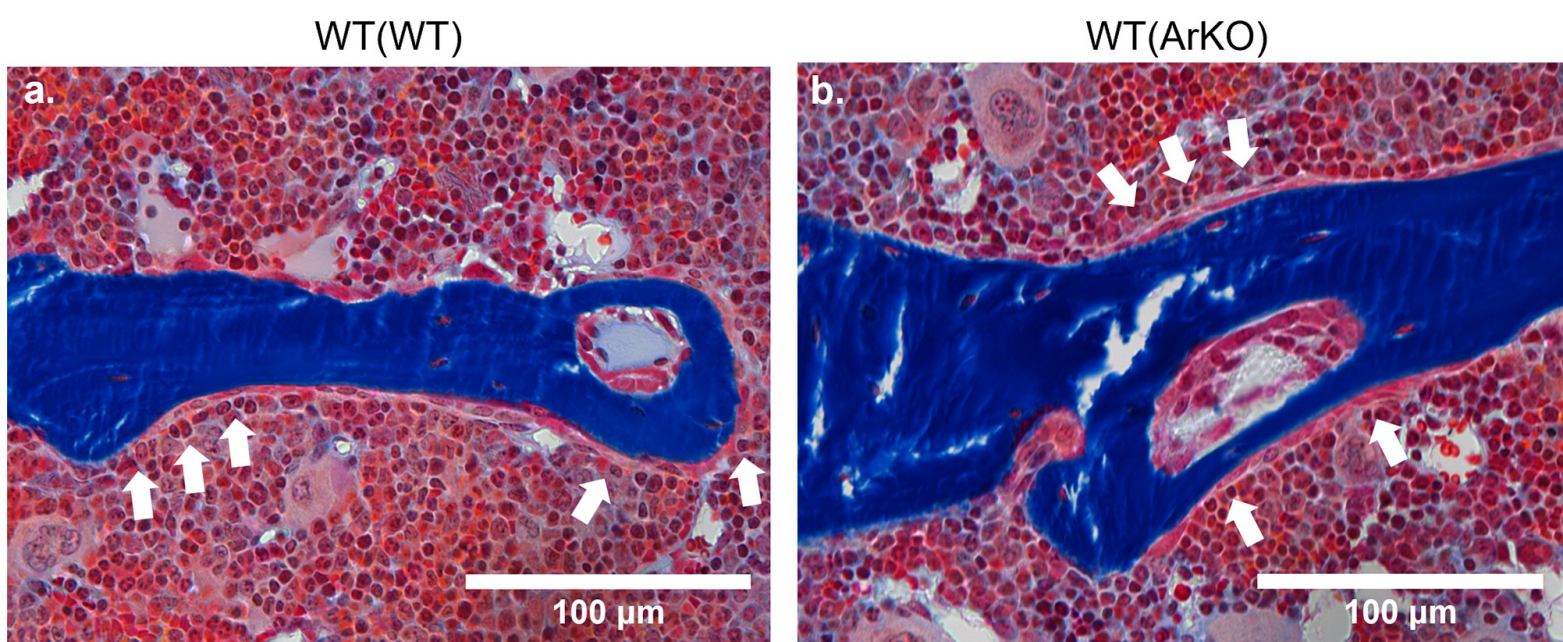
Static histomorphometry bone formation stain shows similar osteoblast activity on the bone surface of WT(WT) and WT(ArKO) mice. Modified Goldner’s Trichrome stained static histomorphometry images of L4 vertebrae in WT(WT) and WT(ArKO) animals. Images were captured at 40X magnification (scale bar = 100 μm). Positively stained mineralized osteoid appears red and is indicated by arrows. Osteoblasts were counted if they resided along the trabecular surface and retained their cuboidal morphology. (WT(WT) n = 9 animals, WT(ArKO) n = 10 animals).

The number of osteoblasts per millimeter of bone perimeter was reduced in WT(ArKO) mice (Fig 3 & Table 1, *p* = 0.04). There was a trend towards decreased osteoblast surface in WT(ArKO) mice, but it did not reach statistical significance (Table 1, *p* = 0.08).

**Table 1:** Static histomorphometry osteoblast parameters.

Osteoblast Parameter	WT(WT)	WT(ArKO)	p-value
	(*n* = 9)	(*n* = 10)	
Ob.S/BS (%)	21.01 ± 5.75	17.15 ± 6.07	0.08
OS/BS (%)	3.57 ± 2.53	2.97 ± 1.83	0.66
OS(Ob+)/BS (%)	3.09 ± 2.13	2.85 ± 1.75	0.91
N.Ob/O. Pm (#/mm)	731.78 ± 517.04	563.82 ± 254.6	0.78
**N. Ob/B. Pm (#/mm)**	**16.83 ± 4.59**	**13.31 ± 4.36**	**0.04**
N. Ob/ T. Ar (#/mm^2^)	120.53 ± 49.18	105.47 ± 37.42	0.32
OS (mm)	0.87 ± 0.67	0.69 ± 0.37	0.91
Ob.S (mm)	4.99 ± 1.8	4.12 ± 1.34	0.24
OS(Ob+) (mm)	0.76 ± 0.6	0.66 ± 0.34	0.84

Abbreviations used in Table 1 are consistent with those approved by the ASBMR Histomorphometry Nomenclature Committee [21]. Data are expressed as mean ± SD.

### WT(ArKO) mice have decreased osteoclast surface, eroded surface, and comparable osteoclast numbers

**Fig 4:**
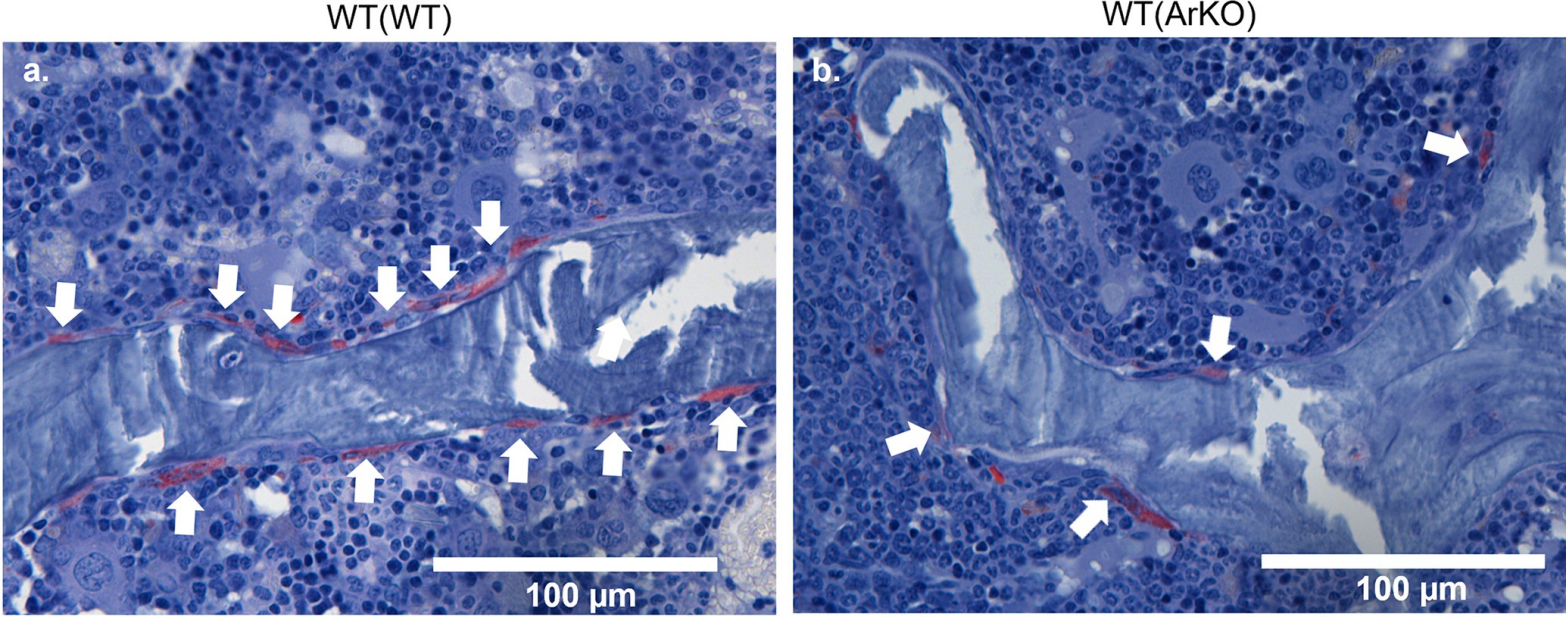
Static histomorphometry TRAP stain shows fewer osteoclasts on the bone surface in WT(ArKO) mice. Static histomorphometry TRAP-stained images of L4 vertebrae in WT(WT) (a) and WT(ArKO) animals (b). Images were captured at 40X magnification (Scale bar = 100 μm). TRAP positive cells appear red and are indicated by the arrows. (WT(WT) n = 9 animals, WT(ArKO) n = 10 animals).

WT(ArKO) animals had significantly decreased osteoclast surface per bone surface (Table 2, *p* = 0.040), fewer positively stained osteoclasts on eroded regions per bone surface (Fig 4 & Table 2, *p* = 0.040) and decreased number of osteoclasts normalized to bone perimeter (Table 2, *p* = 0.014). There were no differences in overall erosion surface, osteoclast surface or osteoclast-positive erosion surface (Table 2).

**Table 2:** Static histomorphometry osteoclast parameters.

Osteoclast Parameter	WT(WT)	WT(ArKO)	p-value
	(*n* = 9)	(*n* = 10)	
**Oc.S/BS (%)**	**14.41 ± 2.94**	**11.27 ± 3.72**	**0.04**
ES/BS (%)	16.58 ± 3.68	13.32 ± 4.57	0.09
**ES(Oc+)/BS (%)**	**14.41 ± 2.94**	**11.27 ± 3.72**	**0.04**
N.Oc/E. Pm (#/mm)	42.77 ± 3.35	40.58 ± 2.61	0.11
**N. Oc/B. Pm (#/mm)**	**6.98 ± 1.11**	**5.34 ± 1.7**	**0.01**
N. Oc/T. Ar (#/mm^2^)	50.37 ± 11.56	44.69 ± 18.51	0.55
ES (mm)	3.95 ± 0.93	3.33 ± 1.37	0.49
Oc.S (mm)	3.43 ± 0.78	2.81 ± 1.12	0.22
ES(Oc+) (mm)	3.43 ± 0.78	2.81 ± 1.12	0.22

Abbreviations used in Table 2 are consistent with those approved by the ASBMR Histomorphometry Nomenclature Committee [21]. Data are expressed as mean ± SD.

### Dynamic histomorphometry results

**Fig 5:**
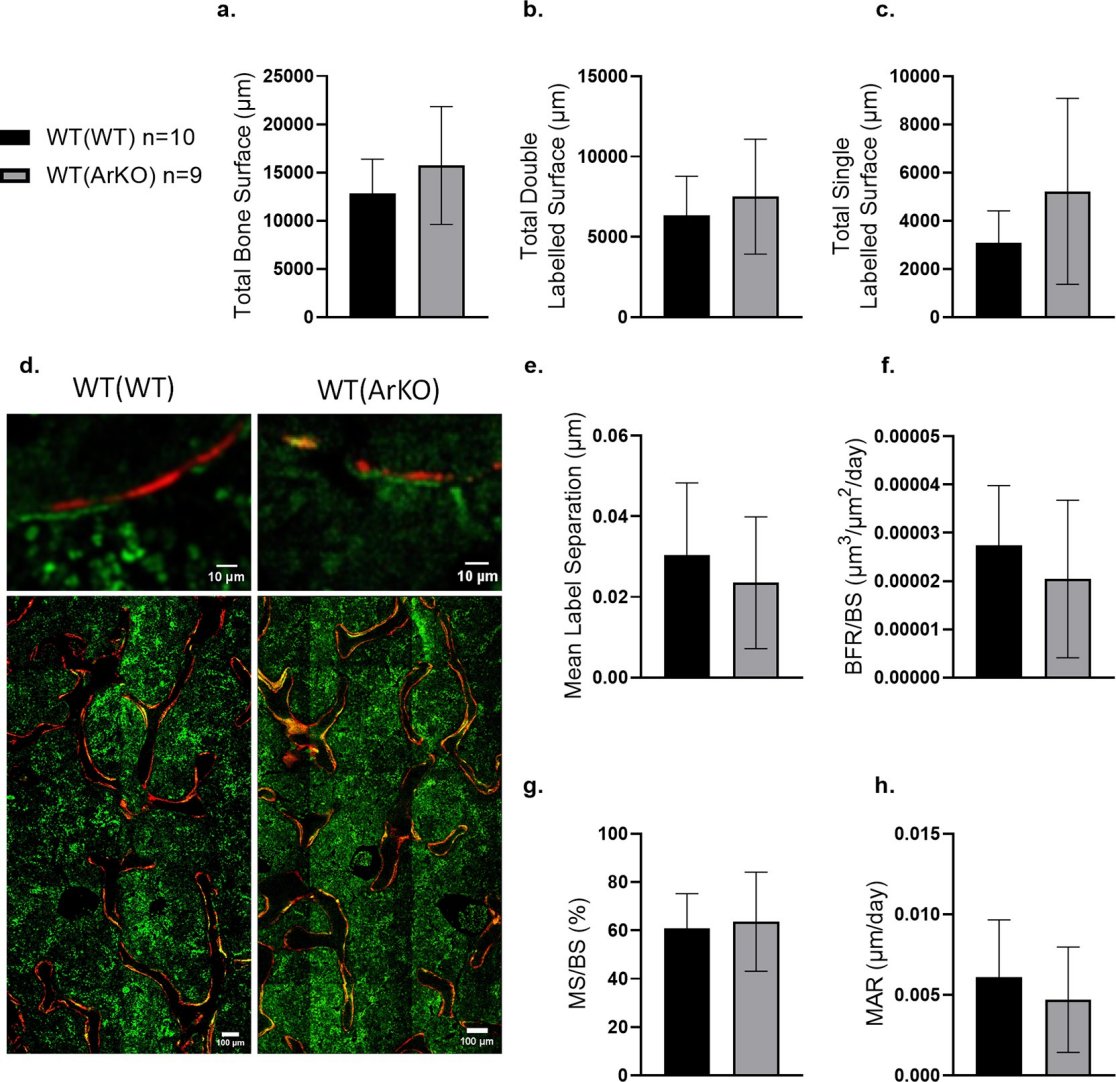
Dynamic histomorphometry of WT(WT) and WT(ArKO) mice. Dynamic histomorphometry of label analysis. Direct measurements included mean total labelled bone surface (a), mean double labelled surface (b), and mean single labelled surface (c). Confocal images (d) were captured at 20X magnification (Top Row scale bars = 10μm, bottom row scale bars = 100 μm). No differences were seen in mean label separation (e), bone formation rate per bone surface (BFR/BS) (f), mineralizing surface per unit bone surface (MS/BS) (g), or Mineral Apposition Rate (MAR) (h). Data are expressed as mean ± SD. (WT(WT) n = 10 animals, WT(ArKO) n = 9 animals).

Direct measurements including total bone surface (Fig 5A), total double labelled bone surface (Fig 5B, *p* = 0.45), and total single labelled (Fig 5C) were not statistically different between groups; however, the means of these parameters were higher in the WT(ArKO) group. On the other hand, except for mineralizing surface normalized to bone surface (MS/BS), the dynamic values were generally lower for WT(ArKO) mice samples, including mean separation of the fluorescent labels, bone formation rate per unit of bone surface (BFR/BS), and mineral apposition rate (MAR) (Fig 5D–5H). The total bone surface result is consistent with higher trabecular volume measured by microCT.

### *Ex vivo*, ArKO bone marrow derived cells form fewer alkaline positive colony forming units and mineralized nodules, but comparable numbers of osteoclasts

**Fig 6:**
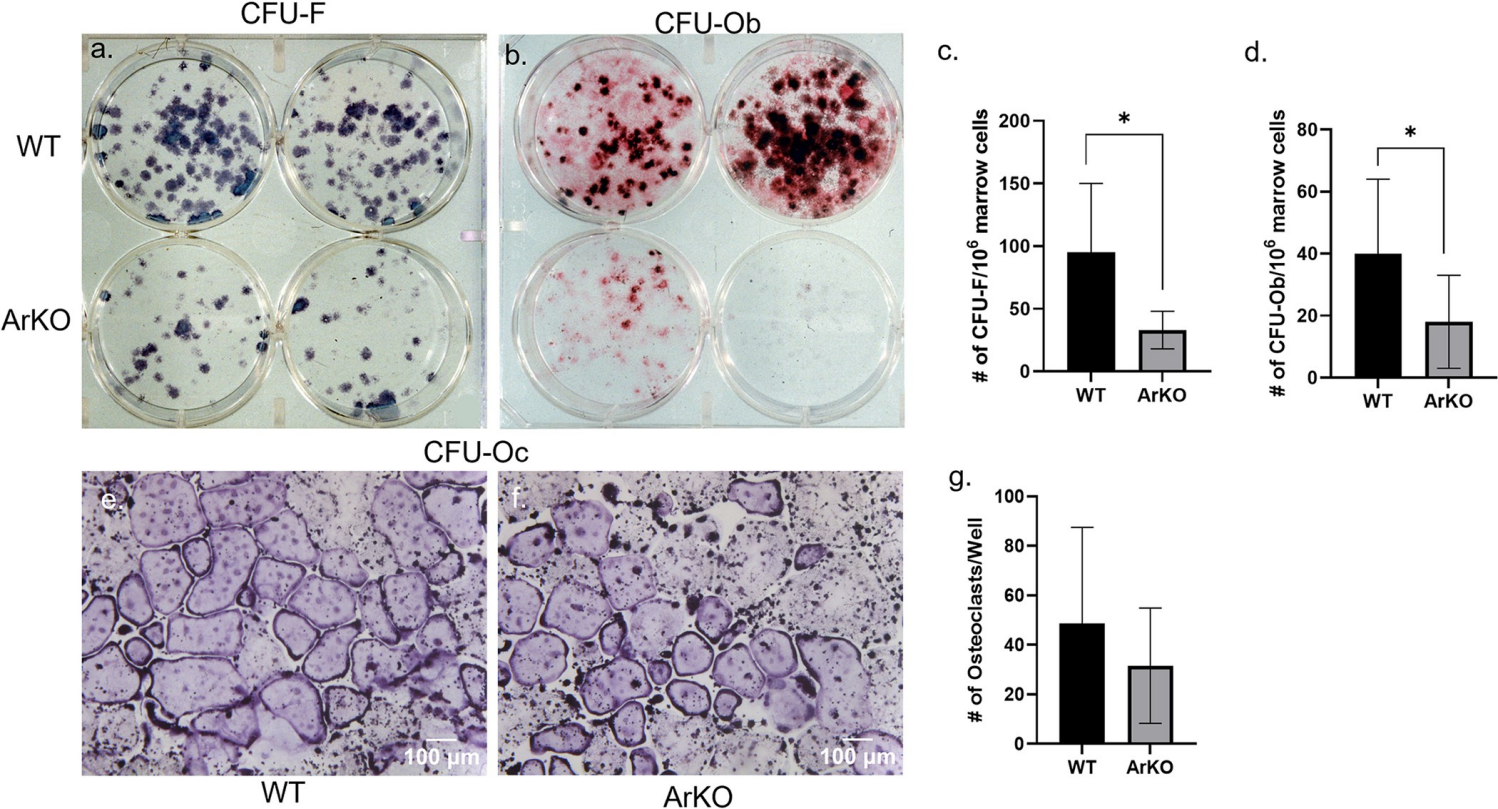
Bone marrow progenitor cells from ArKO mice have deficient formation of early osteoblast precursors (CFU-F) and committed osteoblasts (CFU-Ob), but normal osteoclasts maturation (CFU-Oc). Ex vivo differentiation of primary bone marrow cells from WT (top row) and ArKO (bottom row) animals. Colony counts were decreased in alkaline phosphatase stained ArKO marrow on CFU-F (a & c) (95 ± 55/10^6^ WT marrow cells vs. 33 ± 15/10^6^ ArKO marrow cells, *p* < 0.03) as well as alizarin red stained mineralized nodules (CFU-Ob) (b & d) (40 ± 24 nodules/10^6^ WT marrow cells vs. 18 ± 15 nodules/10^6^ ArKO marrow cells, *p* < 0.03). However, WT (e) and ArKO (f) mice had a similar number of TRAP positive multinucleated osteoclasts/well (49 ± 37 osteoclasts/well vs. 32 ± 23 osteoclasts/well) (g). The results are representative of 3 independent experiments, each experiment using pooled cells from 2 animals per genotype. Osteoclast images were captured at 10X magnification (Scale bar = 100 μm).

Because the histomorphometry results suggested an osteoblast phenotype in WT(ArKO) we undertook studies to determine whether there was abnormal differentiation of osteoblast or osteoclast precursors in the ArKO bone marrow derived cells. Under *ex vivo* differentiation, marrow cells derived from global ArKO mice developed fewer alkaline phosphatase positive fibroblast colony-forming units (CFU-F) (Fig 6A & 6C) compared to WT cells (33 ± 25 no./10^6^ marrow cells (bottom wells) vs. 95 ± 55/10^6^ marrow cells (top wells), *p* < 0.03). ArKO osteoblast colony-forming unit (CFU-Ob) cultures had fewer alizarin-stained mineralized nodules (18 ± 15 no./10^6^ marrow cells (bottom wells) vs. 40 ± 24 no./10^6^ WT marrow cells (top wells), *p* < 0.03), indicating poorer mineralization in the cell autonomous *ex vivo* environment (Fig 6B & 6D). This data suggests either there are fewer osteoblastic precursors or precursors have lower survival in the *ex vivo* conditions and fewer mature osteoblasts, resulting in reduced mineralization capabilities. However, WT (Fig 6E) and ArKO (Fig 6F) under M-CSF and RANKL stimulation, CFU-Oc cultures showed a comparable number of TRAP positive, multinucleated osteoclasts (49 ± 37 osteoclasts/well vs. 32 ± 23 osteoclasts/well, *p =* 0.21) (Fig 6G). Together, this data suggests that male ArKO mice bone marrow cell progenitor pools have a problem in cell autonomous Ob differentiation, but normal cell autonomous Oc differentiation.

## Discussion

In humans and rodents, circulating estrogens are major regulators of bone mass. Previous observations of global aromatase deficiency in humans [3, 25, 26] and mouse models [4, 27] have demonstrated low bone mass and abnormal bone turnover. However, these studies do not distinguish the impact of estrogens produced in the gonads or extra-gonadal sites. Here, we find that ArKO BMC transplant recipients (WT(ArKO) mice) have greater trabecular bone quantity and quality compared to WT BMC transplant recipients (WT(WT) mice). Furthermore, WT(ArKO) animals have smaller cortical pores and reduced porosity which is associated with improved cortical strength [28]. Histologically, WT(ArKO) bone shows fewer osteoblasts and osteoclasts on the bone surface and lower osteoblast and osteoclast surfaces. While there are no statistically significant differences in individual parameters on dynamic histomorphometry, we report greater labelled trabecular surface due to higher bone volume, but with less dynamic formation and mineral apposition on those surfaces. This data describes the net gain in trabecular bone volume due to lower osteoclast activity combined with few changes in osteoblastic parameters.

By dynamic histomorphometry, all animals exhibit very low bone turnover and many overlapping/closely spaced labels. Several factors may be at play including reduced turnover due to age or the inability of bone cells to recuperate after irradiation [29–31]. Directly measured bone surface parameters are lower (single and double labelled surface, BS) in WT(WT) animals, presumably due to higher overall trabecular volume in WT(ArKO). Since osteoblasts are known to regulate osteoclast differentiation, conceivably the low osteoblast number generated the reduced osteoclast surface and activity. Supporting this in *ex vivo* differentiation assays, we observed defective osteoblast differentiation and differentiated function (mineralized nodule formation) without differences in *ex vivo* osteoclast generation from precursors in the ArKO bone marrow cell pool. While these assays don’t recapitulate the environment of a WT host, they do show defective osteoblastic differentiation that could contribute to decrease in the osteoclastic parameters.

Although WT(ArKO) animals share the low bone turnover and reduced osteoblast function features we previously reported in global ArKO animals [4, 18], global ArKO animals have distinct phenotype metabolic and bone phenotypes. Rubinow et al. reported no differences in plasma estradiol levels, no gain in body weight, no changes in body composition, and improved glucose tolerance in WT(ArKO) mice compared to WT(WT) animals [17]. In further contrast to the WT(ArKO) model, global ArKO animals have low trabecular bone volume and trabecular thickness in the axial and appendicular skeleton [4, 18]. This difference between WT(ArKO) and global ArKO mice may result from the inherent difference that global ArKO animals are aromatase deficient from conception, whereas the WT(ArKO) only experienced changes at time of transplantation. Alternatively, our animals received bone marrow cells which are comprised of many cell types including fully differentiated cells and stem cells. Finally, while the transplanted cells contain hematopoietic and mesenchymal stem cells, the stromal, endothelial, and neuronal cell populations, as well as other organs, in the transplant recipients are WT cells whereas in the global ArKO model these cells are ArKO cells.

The cell(s) responsible for the WT(ArKO) phenotype is(are) unclear. Aromatase is expressed in several cell types of hematopoietic and nonhematopoietic origin. For example, previous studies in mesenchymal stem cells (MSCs) isolated from post-menopausal women have shown leptin and vitamin D induced aromatase expression and activity [32]. Several cell types of hematopoietic origin express aromatase including T lymphocytes [12], B lymphocytes [13, 14], and macrophages [15]—the latter of which also express estrogen receptors [17, 33]. Each of these cell types can indirectly regulate the formation and activity of osteoclasts and osteoblasts through the release of soluble factors such as TNFα and RANKL [34]. In our study, these cells in WT(ArKO) animals lacked aromatase which could affect the release of soluble factors and lead to the dysregulation of bone turnover. Our observations may also be partially due to the paracrine signaling of marrow-synthesized estrogen on macrophage populations. Estradiol blocks the expression of macrophage colony-stimulating factor (M-CSF) [35, 36]—the primary regulator of macrophage survival, proliferation, and differentiation—leading to reduced myelopoiesis [37, 38]. Macrophages differentiate from monocytes directly in the bone marrow or from monocytes released into the circulation where they differentiate into tissue-specific macrophages [39]. Since our WT(ArKO) model exhibits a local estrogen deficiency, rather than systemic, monocyte differentiation might have been preserved to influence the high trabecular bone phenotype.

In agreement with our observations of WT(ArKO) mice, a previous study of 14-month-old ERαKO males reported greater trabecular bone volume, greater trabecular number, and decreased trabecular separation within the axial and appendicular skeletons [40]. Moreover, the number of osteocytes expressing sclerostin—a protein which inhibits Wnt signaling and bone formation through paracrine interactions with osteoblasts and osteoclasts—is increased in the cortical bone of ERαKO animals but not in trabecular bone of the femur or lumbar vertebrae [40]. Our study did not address Wnt signaling in either trabecular or cortical compartments, however, this previous study suggests a complex interaction between the bone marrow compartment, transplantable cell populations, and other compartments in bone.

Androgens, like estrogens, are regulators of bone mass in mice and humans [41]. They may act directly or indirectly after peripheral aromatization. Studies of global androgen receptor (AR) knockout (ARKO) have shown the importance of androgens in the preservation of cortical bone mass and stimulation of periosteal apposition [42]. One study of intact ARKO and untreated orchidectomized WT male mice reported a comparable degree of trabecular bone loss compared to sham operated WT mice. When orchidectomized mice were treated with a hormone that cannot be aromatized (dihydrotestosterone (DHT)) or a hormone which can be aromatized to estradiol (testosterone), they reported a loss of trabecular bone mass by pQCT. Furthermore, periosteal mineralization and periosteal bone formation were prevented in WT, but not ARKO mice. Notably, maximal periosteal response in WT mice required aromatization. Together, this suggests androgens can regulate cortical and trabecular bone through the AR without requisite aromatization [42]. Although this study was suggestive of independent androgen effects, the authors did not quantify ERs or aromatase in the bone tissue of these models. Other studies have investigated the regulatory role of androgens in bone remodeling in sexually mature male mice. For example, a study by Matsumoto et al. showed that orchiectomized ArKO males had more severe bone loss and bone resorption than orchiectomized WT mice. This suggests that in sexually mature male mice, androgens and estrogens individually regulate bone mass by suppressing bone resorption. The authors concluded that prepubertal male mice supplemented with androgens require gonadal androgens for the regulation of bone formation during puberty [43]. However, this study had technical limitations in histological evaluations which compromise the conclusions that can be drawn [26]. Studies of human congenital aromatase deficiency reveal a role for estrogens in peak bone mass attainment and periosteal expansion. When an adult male with congenital aromatase deficiency is treated with estrogen supplementation, peak bone mass was attained, and the growth plates closed [25]. In this regard, there was both a decrease in bone resorption and an anabolic effect on bone mass. Estrogen supplementation of a 17 y/o male with congenital aromatase deficiency demonstrated that estrogens are essential for pubertal periosteal expansion associated with the male skeleton and androgens alone are insufficient [44]. In a study of eugonadal men with osteoporosis, Anderson et al. showed that systemic treatment with testosterone was associated with suppressed bone turnover with greater changes in markers of bone resorption than formation. There was a concomitant increase in BMD of the lumbar spine that was more associated with serum estrogen levels rather than serum testosterone [45]. Collectively, these results show androgens and estrogens play a role in the establishment of peak bone mass and regulation of bone turnover pattern in males. Our study established a role for either local bone conversion of androgens into estrogens or local production of estrogens in regulating local bone turnover and osteoblast surface.

Our study has some limitations. First, we did not have a sham transplant control group. We do not expect a major phenotype would have been observed in the sham group, but it would have been a control for any stress related to mock irradiation and blood draws. Secondly, we did not have ArKO transplant recipients to generate ArKO(WT) and ArKO(ArKO) experimental groups. The addition of ArKO transplant recipient groups could help to define the role of estrogens derived from bone resident cells on trabecular bone and cortical bone, particularly stromal cells, but lack of it does not detract from the observed phenotype of WT(ArKO) mice. Thirdly, while we do have previous evidence of plasma estradiol levels from Rubinow et al. [17], we did not obtain plasma testosterone level measurements. Circulating testosterone could be a substrate for aromatase in bone. Finally, we did not obtain measurements of the appendicular skeleton, which may exhibit different phenotypes than that of the axial skeleton [46–48].

## Conclusions

This study shows greater trabecular bone quantity and quality (connectivity, mineralization) and cortical porosity when aromatase deficient bone marrow cells are transplanted into WT mice. There was a reduction in osteoblastic and osteoclastic parameters, with a more pronounced osteoclastic phenotype at the tissue level. Cell autonomous osteoblast in vitro differentiation studies using marrow cells showed defective differentiation of ArKO derived cells. Therefore, hematopoietic cell or transplanted MSC derived aromatase expression is important in maintaining bone in male mice. The exact cell lineage(s) responsible for the observed phenotype in WT(ArKO) mice remains to be defined. Since cells derived from the hematopoietic compartment may migrate out of the bone marrow and cells migrate into the bone marrow compartment, we are not able to state specifically whether the observed phenotypes result from cells residing only in the local bone microenvironment or pathways acting outside the skeleton. Future studies could delete aromatase from specific subpopulations of bone marrow cells to potentially identify the cell lineage most responsible for the low turnover state we observed. These cells may also regulate stem cell allocation which could explain why our cell autonomous in vitro studies showed decrease in osteoblast colony formation and mineralized nodule formation.
